# Atomistic Modeling of the Negative Thermal Expansion in *δ*-Plutonium Based on the Two-State Description

**DOI:** 10.3390/ma5061040

**Published:** 2012-06-07

**Authors:** Tongsik Lee, Michael I. Baskes, A. C. Lawson, Shao Ping Chen, Steven M. Valone

**Affiliations:** 1 Los Alamos National Laboratory, Los Alamos, NM 87545, USA; E-Mails: baskes@lanl.gov (M.I.B.); aclawson@cybermesa.com (A.C.L.); sc@lanl.gov (S.P.C.); smv@lanl.gov (S.M.V.); 2 Jacobs School of Engineering, University of California, San Diego, La Jolla, CA 92093, USA

**Keywords:** plutonium, negative thermal expansion, Invar, Weiss model, modified embedded atom method, Monte Carlo method

## Abstract

The *δ* phase of plutonium with the fcc structure exhibits an unusual negative thermal expansion (NTE) over its narrow temperature range of stability, 593–736 K. An accurate description of the anomalous high-temperature volume effect of plutonium goes beyond the current capability of electronic-structure calculations. We propose an atomistic scheme to model the thermodynamic properties of *δ*-Pu based on the two-state model of Weiss for the Invar alloys, inspired by the simple free-energy analysis previously conducted by Lawson *et al*. The two-state mechanism is incorporated into the atomistic description of a many-body interacting system. Two modified embedded atom method potentials are employed to represent the binding energies of two competing electronic states in *δ*-Pu. We demonstrate how the NTE takes place in *δ*-Pu by means of Monte Carlo simulations implemented with the two-state mechanism.

## 1. Introduction

Plutonium has six equilibrium solid phases at atmospheric pressure. The fcc phase of plutonium called *δ*-Pu exhibits an unusual negative thermal expansion (NTE) over its narrow stability range at high temperatures, 593–736 K. This phase is also known for its wide range of macroscopic anomalies, such as extraordinarily high elastic anisotropy, largest atomic volume (albeit the only close-packed structure among the allotropes), strong elastic softening at elevated temperature, and extreme sensitivity to dilute alloying. In spite of considerable advances in electronic-structure calculations over the past decades, an accurate description of the thermodynamic properties of plutonium goes beyond the current capability of the approach. Monte Carlo (MC) simulations combined with an effective interatomic potential can be an excellent alternative to study the thermodynamic equilibrium of materials from the atomistic perspective. While many suggestions have been put forward to understand the atomic mechanisms for the NTE of materials, those that rely solely on large transverse vibrational amplitudes (such as the “tension” effect [1], or the “rigid unit modes” [2]) are inappropriate to account for the NTE of the bulk close-packed structures at high temperatures [3]. Hence, we must look for a non-vibrational cause for the anomalous thermal expansion of *δ*-Pu.

The iron-based Invar alloys are perhaps the best known examples of simple fcc crystals with unusual thermal expansion. The term “Invar” is originally linked to the nearly “invariable” thermal expansion observed in the Fe-Ni system of 35 at.% Ni near room temperature, first discovered by Guillaume [4], but it is often used generically to refer to iron-rich alloys showing abnormal temperature variations in volume [5]. Weiss [6] proposed a simple model to explain the anomalous thermal expansions of the Invar alloys, which postulates that fcc iron can exist in two magnetic states, closely separated in energy: an antiferromagnetic state with a low magnetic moment and a ferromagnetic state with a high magnetic moment. In this model, it is the excess entropy associated with the thermal excitations between the two magnetic states that is responsible for the unusual thermal expansion of these alloys. The existence of such stable, nearly degenerate stable magnetic states in fcc iron was later supported by density-functional theory calculations [7,8]. Although the Weiss model is phenomenological in nature (in the sense that it does not require the exact details of magnetism but only two competing energy states), it has achieved remarkable success in describing a wide variety of characteristic thermodynamic behaviors of iron and its alloys, including their phase stabilities as well as thermal expansions [9,10,11,12,13].

An analogy to the Invar alloys has been constructed for *δ*-Pu in the course of accumulating knowledge about its electronic structure. Johansson suggested that the 5*f* electrons in Pu straddle the transition from itinerant (conductive, bonding) to localized (magnetic, chemically inert) states in the actinide series [14]. This feature is also ingeniously indicated in the rearranged periodic table by Smith and Kmetko [15], where the elements in the *d* and *f* electron series are diagonally divided into two categories with either itinerant or localized electrons; however, some elements along the diagonal, such as Fe and Pu, belong to neither of these categories because of their elusive electronic nature. With regard to the 5*f* series, the light actinides up to Pu are characterized by the itinerant 5*f* electrons, while in the heavier actinides those electrons tend to be localized. Eriksson *et al*. [16] described the transition from the itinerant to localized electronic behavior as progressively taking place within Pu, and the *δ* phase encompasses a variety of competing electronic configurations in both itinerant and localized states. The cause of the characteristic thermodynamic properties of *δ*-Pu analogous to the Invar alloys was anticipated in their study. The presence and role of magnetism in plutonium are subjects of great controversy [17,18,19,20,21,22,23,24]. As remarked above, however, the application of the Weiss two-state model is not restricted to magnetic systems. Indeed, Lawson *et al*. [25,26] adopted the effective two-state picture to describe the anomalous temperature dependence of the thermodynamic properties in the *δ* phase of Pu and Pu-Ga alloys in terms of a simple free-energy model. Although a full understanding of the electronic nature of plutonium remains a major challenge, we may hold an interpretation of the two states present in the *δ* phase as a competition between electrons with different degrees of delocalization, as suggested in References [16,22,26,27,28,29,30].

In the present study, we develop a novel atomistic scheme to model the *δ* phase of elemental plutonium and demonstrate how its NTE takes place at high temperatures. We employ two sets of modified embedded atom method (MEAM) potentials [31] to represent the interaction between the Pu atoms of distinct electronic states in the fcc structure in accordance with the Weiss picture. The idea behind the present scheme is inspired by the aforementioned approach taken by Lawson *et al*. [25,26]. While the simple isotropic free-energy model in Reference [26] had a striking success in explaining the unusual temperature variation of the thermal expansion and stiffness, the present atomistic modeling relying on effective interatomic potentials is more general in describing the full equation of state for a solid, in particular its sensitivity to shear deformations, as well as to the creation of defects, which is one of key elements for simulating radiation damage effects [32,33,34]. In Section 2, we first discuss a scheme to incorporate the Weiss two-state mechanism into the MC simulation of a many-body interacting system. We then demonstrate in Section 3 that anomalous thermal expansion of *δ*-Pu can be correctly modeled by means of the MC simulation implementing the two-state mechanism. Finally, conclusions and remarks are given in Section 4.

## 2. Simulation Methods

### 2.1. Two-State Model Description of Many-Body Interacting Systems

Despite the appealing simplicity of the Weiss model, the idea has rarely been discussed in the context of atomistic modeling. A notable exception is the work by Gruner *et al*. [35], in which the Weiss picture is employed to model the characteristic thermoelastic properties of the Invar alloys. In this approach, utilizing two Lennard–Jones (LJ) potentials assumed to represent the low- and high-magnetic moment states, the magneto-volume coupling is incorporated by switching from one LJ potential to the other, according to local-moment alignments determined by an Ising-like interaction. Although this study was able to demonstrate the non-trivial thermodynamic behaviors of the Invar alloys, the pairwise LJ potential is too simple to account for the details of the complex properties inherent to metallic systems. More recently, Yokoyama and Eguchi [36] explored a similar idea in their study of the low-temperature thermal expansion of the Fe-Ni Invar alloy by means of embedded atom method (EAM) potentials.

Plutonium is a highly covalent material due to the strong directionality of the *f*-electron orbitals, and therefore we employ the MEAM potential [37,38,39] to capture the angular dependence of electron densities. Baskes [31] previously developed a MEAM potential that reproduces numerous features of the element. The details of the MEAM formalism have been extensively discussed in the literature. Below we develop an MC scheme to incorporate the two-state mechanism into atomistic modeling similar to the approach pursued in Reference [36] but discuss the background rationale that was not addressed in the reference. Specifically, we discuss the two vital elements in implementing MC simulations: a statistical ensemble to simulate the system, and the form of the probability density pertinent to the ensemble.

**Figure 1:**
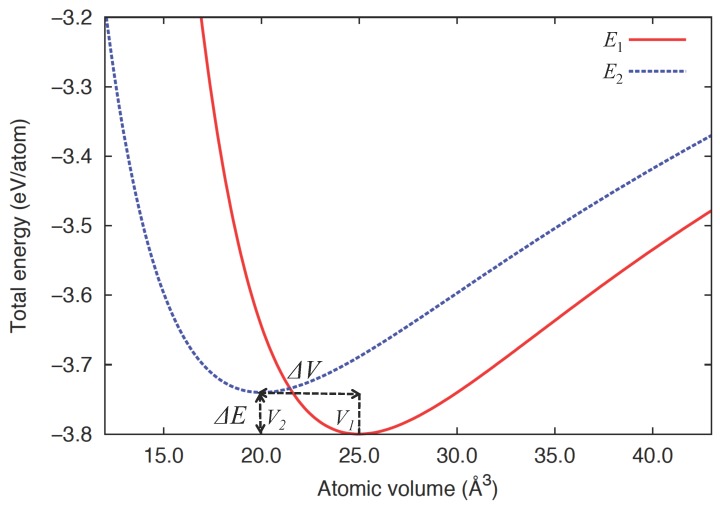
Binding energy curves $E_{1}\left(V\right)$ and $E_{2}\left(V\right)$ for the two competing electronic states in an effective two-state description of *δ*-Pu. ΔV is the difference between the equilibrium volumes $V_{1}$ and $V_{2}$, while ΔE (=$E\left(V_{2}\right)-E\left(V_{1}\right)$) is the energy separation at equilibrium for the two states.

In the two-state model description, we assume that *δ*-Pu is effectively characterized by two competing binding energy curves corresponding to distinct electronic states, in line with first-principles studies [16,22,25,26,27,28,29,30]. We henceforth refer to these electronic states as States 1 and 2 for simplicity. Thus, the total energy of this system is no longer a single-valued function of a given configuration, and each of the energy states $E_{1}$ and $E_{2}$ individually depends on volume. (The approach taken here may be contrasted with the two-band model [40,41], where the mixtures of different bands result in a single electronic surface, upon which molecular dynamics is performed. In the present approach, there are two separate electronic states that individually couple to an atomic configuration.) In accordance with the Invar alloys, we consider that State 1 is lower in energy but higher in equilibrium volume than State 2, by ΔE and ΔV, respectively. The energy-volume relations of these states are illustrated in Figure 1. The system in this description is not essentially different from a simple binary alloy if we interpret the atoms in each state as those with distinguishable “chemical identity” or “type”, as appropriately coined “self-intermetallic” by Lawson *et al*. [42]. Here, the state (or type) of the atoms is subject to change from one to the other under the influence of thermal agitation or chemical alloying, such that the number of the atoms in each state $N_{1}$ and $N_{2}$ is not held fixed but varies, while the total number of atoms *N* is conserved.

To implement MC simulations, a statistical ensemble must first be specified. Here, we wish to simulate the system at constant external pressure *p* and temperature *T*, so as to find the equilibrium volume and fraction of the atoms in each state at those *p* and *T*. Such an ensemble is known as the semi-grand ensemble [43,44]. In order to find the condition for the thermodynamic equilibrium, consider the Gibbs free energy per atom, G(x), as a function of fraction x(=$N_{1}/N)$ of the atoms in State 1,

(1) $$G\left(x\right)=x\mu_{1}+\left(1-x\right)\mu_{2}$$

where $\mu_{1}$ and $\mu_{2}$ are the chemical potentials of states 1 and 2, respectively. The equilibrium condition follows directly from the Gibbs–Duhem relation:

(2) $${\left(\frac{\partial G(x)}{\partial x}\right)}_{N_{1}+N_{2},\;p,\;T}=\mu_{1}-\mu_{2}=0$$

*i.e.*, the relative chemical potential between States 1 and 2 is set to be zero. In some common applications of the semi-grand ensemble such as free surfaces of a binary alloy [45], the relative chemical potential is specified such that the bulk-like composition of the alloy is overall maintained. In contrast, we set the relative chemical potential to zero in the present context in order to let the system optimize its composition at equilibrium. Thus, a trial move where an atom changes its identity can be performed as though the factor involving the relative chemical potential is irrelevant in the Metropolis acceptance rule discussed below.

A certain ambiguity seems to be involved in the question of how the two-state picture can be incorporated into a system with many-body interactions. Recall that the original context of the Weiss model postulates that the system is essentially described as a simple Schottky two-level system, where *N* independent atoms individually take either of two energy levels $E_{1}$ and $E_{2}(>$$E_{1}$), separated from each other by ΔE. The *N*-particle partition function $Z_{N}$ is thus a product of one-particle partition functions $Z_{1}$ of each atom:

(3) $$Z_{N}={\left(Z_{1}\right)}^{N}={\left(g_{1}\;e^{-\beta E_{1}}+g_{2}\;e^{-\beta E_{2}}\right)}^{N}$$

where *β*=${\left(k_{B}T\right)}^{-1}$ ($k_{B}$ is the Boltzmann constant), and $g_{1}$ and $g_{2}$ are the degeneracies of States 1 and 2, respectively. These parameters can be determined from the values of magnetic moments in the case of the Invar alloys [6]. In reality, the atoms in solids are not independent but interact with each other through interatomic forces, hence Equation (3) cannot be utilized as it is when a system consisting of interacting atoms is simulated.

Let us denote by $\left\{r_{l}\right\}$ an atomic configuration $\left\{r\right\}=\left\{r_{1},r_{2},\ldots ,r_{N}\right\}$ with a configuration of states (or types) $\left\{l\right\}=\left\{l_{1},l_{2},\ldots ,l_{N}\right\}$, each of whose components is either 1 or 2 for a two-state (or binary) system. In the EAM-type models, including the MEAM, the total energy *E* of a system for a given configuration $\left\{r_{l}\right\}$ is given by

(4) $$E\left(\{r_{l}\}\right)=\sum_{i=1}^{N}\left[F_{l_{i}}\left[\bar{\rho_{i}}\right]+\frac{1}{2}\sum_{j\neq i}\Phi_{l_{i}l_{j}^{\prime }}\left(r_{ij}\right)\right]$$

here, $F_{l_{i}}$ is the energy to embed an atom *i* in the state $l_{i}$ into its host electron density ${\bar{\rho }}_{i}$, and $\Phi_{l_{i}l_{j}^{\prime }}\left(r_{ij}\right)$ is a pair interaction between the *i*-th and *j*-th atoms, respectively in the states $l_{i}$ and $l_{j}^{\prime }$, separated by $r_{ij}$. In common with any *N*-scaling energy expression, *E* in the EAM-type models can be written as a sum over the energies of individual atoms $E_{l_{i}}(=$$F_{l_{i}}+1/2\sum_{j\neq i}\Phi_{l_{i}l_{j}^{\prime }})$:

(5) $$E\left(\{r_{l}\}\right)=\left(\sum_{i=1}^{N}E_{l_{i}}\right)\left(\{r_{l}\}\right)$$

even though the energy of each individual atom depends on the configuration of the environmental atoms. We propose to express the *N*-particle partition function in the present context by analogy with Equation (3), such that

(6) $$Z_{N}=\int d^{N}r_{l}\prod_{i=1}^{N}\left[g_{1}\;e^{-\beta E_{l_{i}=1}}\left(\left\{r_{l};l_{i}=1\right\}\right)+g_{2}\;e^{-\beta E_{l_{i}=2}}\left(\left\{r_{l};l_{i}=2\right\}\right)\right]$$

where $\left\{r_{l};l_{i}\right\}$ stands for a configuration $\left\{r_{l}\right\}$ with the *i*-th atom in state $l_{i}$, and the integration is taken over all possible combinations of $\left\{r_{l};l_{i}\right\}$. It follows that the probability to find the system at a configuration $\left\{r_{l};l_{i}\right\}$ is given by

(7) $$\rho \left(\{r_{l};l_{i}\}\right)\propto g_{l_{i}}\;e^{-\beta E_{l_{i}}}\left(\left\{r_{l};\;l_{i}\right\}\right)$$

thus, one can sample the distribution according to the following Metropolis acceptance rule:

(8) $$\mathrm{acc}\left(l_{i}\to l_{i}^{\prime }\right)=\min\left\{1,\frac{g_{l_{i}^{\prime }}\;e^{-\beta E_{l_{i}^{\prime }}}\left(\{r_{l};\;l_{i}^{\prime }\}\right)}{g_{l_{i}}\;e^{-\beta E_{l_{i}}}\left(\{r_{l};\;l_{i}\}\right)}\right\}$$

where a trial move is attempted by switching the state of the *i*-th atom from $l_{i}$ to $l_{i}^{\prime }\;(\neq$$l_{i})$ for a given atomic configuration {r}. Trial moves of this type are implemented in conjunction with those in the scaled-atomic coordinates and the volume of the periodic cell to simulate the full phase space of the isobaric-isothermal ensemble.

### 2.2. Computational Details

We perform classical MC simulations with parallel tempering using 32 independent replicas of isobaric-isothermal ensembles, each of which differs in temperature, ranging from 20 K to 1,300 K, with equal incrementation. The external pressures of all replicas are set to zero. A periodic cell containing 500 atoms is used to represent a fcc single-crystal bulk system at each temperature. The general implementation of the simulations conducted in this study is similar to that previously presented in Reference [46], except that an extra type of trial moves to exchange electronic states (Equation (8)) is included. One of the MC moves among the scaled coordinates, the volume of the simulation cell, or the electronic states is randomly attempted according to certain probability ratios (0.9:0.05:0.05) within each replica. With the aim of accelerating the equilibrium, parallel tempering is executed on a fixed schedule of every 100 MC moves, where atomic configurations are exchanged between adjacent replicas at both lower and higher temperatures in succession. We utilize the method of block averages, where each block consists of a half million trial moves. Data were collected from 20 blocks after discarding the first few blocks. Quoted statistical errors in the presented figures indicate one standard deviation.

With regard to state 1, we adopt the MEAM parameters published in Reference [31], except for a modification to have $t_{3}=0$ [47]. This parameter is correlated with the inversion symmetry in the crystal structure primarily pertinent to the monoclinic *α* phase, and its original value (−0.8) stabilizes the *α* phase over the *δ* phase at low temperatures, in agreement with experiment. This modification is not absolutely necessary in the present analysis of the high-temperature properties of the *δ* phase, but it allows us to examine the overall temperature variations of thermodynamic properties of this phase even outside the actual range of stability. To represent state 2, we slightly modify the potential for state 1 such that it has a higher binding energy and smaller equilibrium volume than state 1, by ΔE=60 meV (∼700 K) and ΔV=4.96 ${Å}^{3}$, respectively. State 2 is also assumed to have slightly smaller stiffness by 30%.

High-temperature experimental volume was selected for the initial volume of the system at all temperatures. All the Pu atoms are initially in state 1 at all temperatures. We determine the cross interaction $\epsilon_{12}$ between the atoms in the two states by introducing the mixing energy Δ, such that

(9) $$\epsilon_{12}=\frac{\epsilon_{1}+\epsilon_{2}}{2}-\Delta$$

where $\epsilon_{1}$ and $\epsilon_{2}$ are the sublimation energies of states 1 and 2, respectively. Allowing Δ to be a free parameter, we investigate the simulation data presented below for various values of the parameter. For convenience, other parameters associated with the interactions between the atoms in the two states are determined by simple arithmetic means. In the absence of knowledge concerning the degeneracies in the two electronic states, the ratio of these parameters is assumed to be unity. Detailed effects of the arbitrarily chosen parameters are not examined in this study.

## 3. Simulation Results

In the analysis given below, we address the temperature dependence of equilibrium properties with a varying degree of segregation between the atoms in the two states. Five values of mixing energy Δ are selected from the range between +0.05 and +0.24 eV. All values are taken to be positive such that the atoms in different states are segregated in the absence of thermal agitation, for the relatively moderate energy separation adopted in this study, $\Delta E/k_{B}∼$700 K, in order to ensure that state 1 is fully occupied at 0 K or sufficiently low temperatures in all cases.

**Figure 2:**
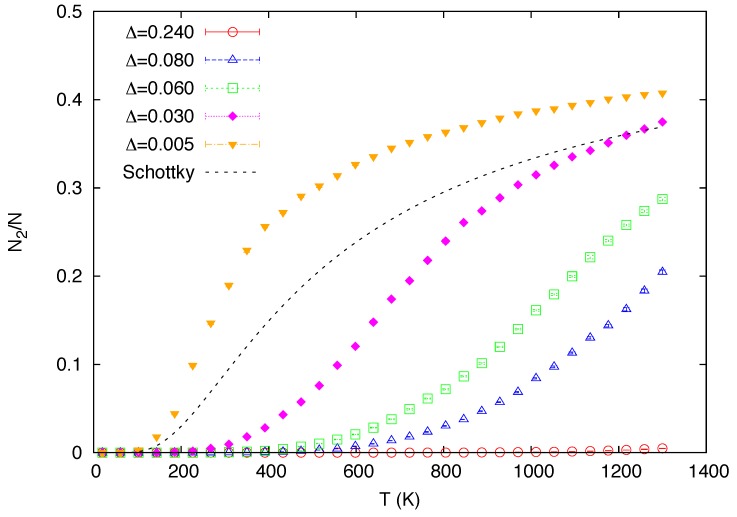
Calculated temperature dependence of the relative occupation of state 2 for a varying value of the mixing energy Δ (in eV). A similar curve obtained from the simple two-level statistics (with $\Delta E/k_{B}∼$700 K and $g_{1}/g_{2}=1$) is also included for reference (labeled as “Schottky”).

The temperature dependence of the relative occupation of state 2 predicted by the simulations is shown in Figure 2. A similar curve directly obtained from the simple Schottky two-level statistics Equation (3) (with $\Delta E/k_{B}∼$700 K and $g_{1}/g_{2}=1$) is also included in the figure for reference (labeled as “Schottky”). While virtually no atoms are excited at all temperatures in the case of the strongest segregation, Δ=0.24 eV, the atoms in state 2 come into the system at finite temperatures as the segregation is weakened by reducing the magnitude of Δ. Hence, all the data for Δ=0.24 eV presented below can essentially be interpreted as what is expected for the simulations with the system consisting of only State 1. Although these curves predicted by the present model are qualitatively similar to that of the simple two-level model, the former shows more complicated dependencies on the parameters in the interatomic potentials, which are absent in the latter.

**Figure 3:**
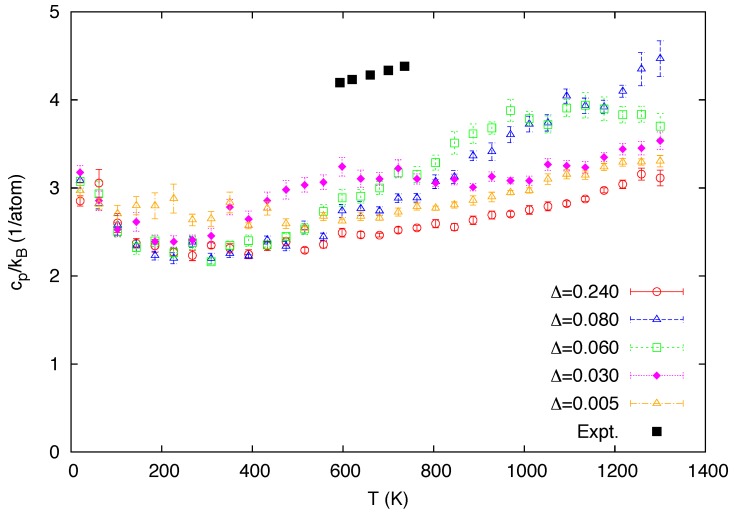
Calculated temperature dependence of the heat capacity per atom, scaled with the Boltzmann constant, for a varying value of the mixing energy Δ (in eV). The experimental data [48] in the stability range of *δ*-Pu are also included for comparison.

In Figure 3, the predicted temperature variations of the isobaric heat capacity per atom ($c_{p}$, scaled by $k_{B}$) for the five mixing energies are compared with the experimental data in the stability range of *δ*-Pu [48]. These heat capacity data, as well as the thermal expansion data discussed next, were obtained from the root-mean square fluctuations of relevant thermodynamic variables [49]. These quantities are associated with the second derivatives of the free energy and hence the calculated data are relatively less converged than the other data presented in this study. While the main focus of the present study is the reproduction of the anomalous behavior of the *δ* phase at high temperatures where classical MC simulations are legitimate, the data computed from the classical simulations should not be taken to be realistic below the Debye temperature (∼120 K for Pu-2 at. % Ga [50]), where both the heat capacity and thermal expansion vanish as temperature approaches absolute zero. Classical simulations by no means capture such behavior governed by quantum effects. The predicted heat capacity data exhibit Schottky-like peaks as the fraction of the atoms in the higher state changes with temperature (Figure 2), similarly to the case of the simple two-level model, in which the excess contribution to heat capacity is directly proportional to the temperature derivative of the fraction [13]. The position of the peaks shifts towards lower temperature as the atoms are excited by lower thermal energy with decreased Δ.

There are at least two reasons for the discrepancy with the experimental data. First, the MEAM potential of Reference [31] adopted in this study exhibits a pronounced dip in the heat capacity curves at low temperatures. This effect reduces their values from what is anticipated from the Dulong–Petit law ($c_{p}/k_{B}∼$3), whereby the overall temperature profile of the heat capacity is lower than the experimental data, even though it is incremented with the excess contribution from the thermal excitation between the two states. The dip is most likely a mere artifact in this particular parametrization of the potential, and is due to neither the intrinsic nature of the MEAM model nor the necessary outcome of the two-state mechanism. Secondly, the conventional interatomic potential models, including the MEAM, neglect any effect of the thermal excitation of electrons to states above the Fermi energy. Experimentally, the electronic contribution to the entropy is substantial, however [48,51]. Although the present model partially accounts for the electronic contributions originating from the mixing of the two states, neither of these states capture the electronic thermal excitation in the above sense.

**Figure 4:**
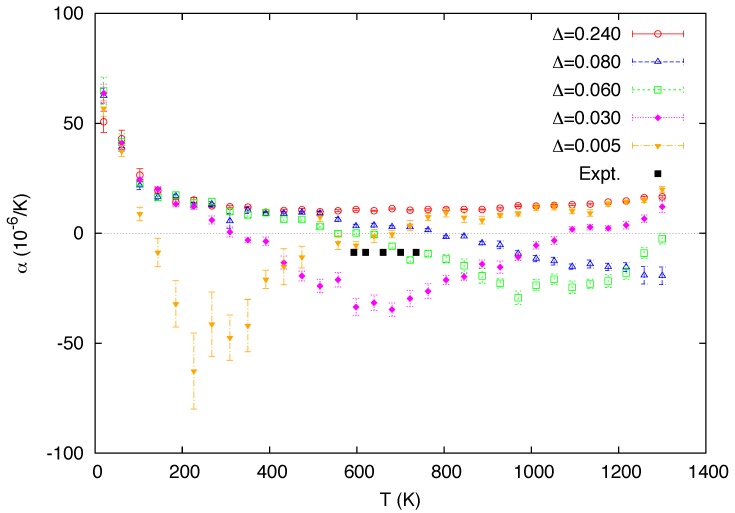
Calculated temperature dependence of the linear coefficient of thermal expansion for a varying value of the mixing energy Δ (in eV). The experimental data [48] in the stability range of *δ*-Pu are also included for comparison.

**Figure 5:**
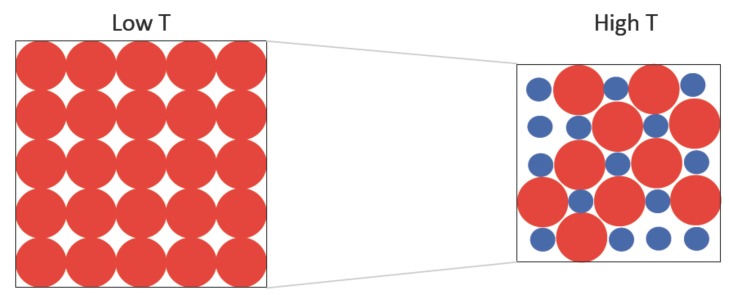
Schematic illustration of how volume contraction occurs in the Weiss two-state picture. The overall volume of the system is reduced as more atoms are excited to the small-volume state at higher temperature. This effect is in competition with the usual volume expansion due to the anharmonic effect of lattice vibrations. The fraction of small-volume atoms is exaggerated for visual effect in this representation.

Figure 4 shows the temperature dependence of the linear coefficient of thermal expansion (CTE) in comparison with the experimental data [48]. The CTE usually exhibits a similar temperature profile to the heat capacity in many materials, but it is not in the general case, especially when there is NTE [52]. It can be seen however that the positions of the negative peaks in the CTE are well synchronized with that of the positive peaks of the heat capacity in Figure 3. In the two-state description, thermal expansion is suppressed when a significant number of atoms are excited to the small-volume state. This effect of volume contraction counteracts the usual anharmonic effect of the lattice vibrations which favors increase in volume. The former effect can override the latter effect when the volume difference between the two states is sufficiently large, such that thermal expansion is driven to a negative value. This mechanism is schematically depicted in Figure 5. The CTE curves predicted from the present simulations are qualitatively similar to those previously described by the free-energy analysis [26], apart from the discrepancies at low temperatures due to the lack of the quantum effect. We note that the unrealistically steep decreases in the calculated CTE curves at very low temperatures are related to the unphysical dip in the heat capacity (cf. Figure 3) discussed in the last paragraph.

**Figure 6:**
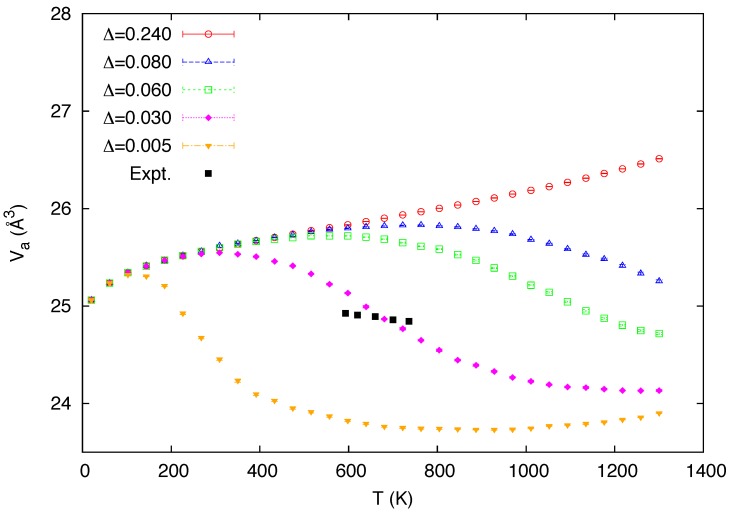
Calculated temperature dependence of the atomic volume for a varying value of the mixing energy Δ (in eV). The experimental data [48] in the stability range of *δ*-Pu are also included for comparison.

The predicted temperature variation of the atomic volume is shown in Figure 6, along with the experimental data [48]. Although it may appear somewhat questionable to predict a high-temperature atomic volume using the MEAM potential (for state 1) whose 0 K volume was determined from the high-temperature experimental volume, first-principles calculations [16,53] predict the 0 K volume at the ground state to be nearly comparable to the experimental value. The curve for Δ=0.03 eV agrees with the experimental values around the middle of the stability range of the *δ* phase, but the negative slope is overestimated in the data, consistent with the excessively negative values of the corresponding CTE data for the Δ seen in Figure 4.

In the original Weiss model relying on the simple Schottky two-level system, the change with temperature in the fraction of the small-volume atoms is not characterized by a phase transition but a crossover. That is, there is no discontinuity in the free energy, or any of its derivatives. Although it is not obvious that this should be the case for the present system consisting of interacting atoms, neither the first derivatives (Figure 2 and Figure 6) nor the second derivatives (Figure 3 and Figure 4) of the Gibbs free energy appear to show any signs of discontinuities, even if a possible finite size effect is taken into consideration.

## 4. Conclusions

We have demonstrated that the unusual high-temperature thermal expansion of *δ*-Pu can be reproduced by the MEAM potential in conjunction with an MC scheme that incorporates the Invar mechanism based on the Weiss two-state picture. While the original Weiss model relies on the simple two-level Schottky description, the mechanism has been generalized to a more realistic system with many-body interactions. The key to the reproduction of the anomalous volume effect is the generation of excess entropy associated with the thermal excitation between two competing electronic states with distinct volume dependence. The simulation results are in qualitative agreement with the previous free-energy analysis based on the Weiss model [26]. In concert with the growing fraction of the small-volume atoms in the energetically higher state with increasing temperature, the heat capacity generates a typical Schottky-like anomaly. The thermal expansion is concurrently suppressed when the atoms in the small-volume state are substantially populated in the system. When the volume difference between the two states is sufficiently large, the effect of the volume contraction outweighs the usual volume expansion due to the lattice anharmonicity so that NTE takes place at finite temperatures.

Owing to the lack of certainty in first-principles results, the model proposed in this study, at least at the present stage of development, involves a certain degrees of arbitrariness, particularly in the determination of the small-volume state and the cross interaction between the atoms in the two states. Nevertheless, it offers a clear atomistic perspective on how competing electronic states present in *δ*-Pu would affect its thermodynamic properties as a function of temperature. In this article we have limited ourselves to discussing only the volume effects of unalloyed *δ*-Pu. The scheme presented in this study is however envisaged to provide a robust analytical basis to address diverse phenomenological aspects of plutonium and its alloys, including elasticity, dilute-alloying effects, and phase stability. A comprehensive discussion of these topics will be given elsewhere [54]. The construction of the reliable atomistic model of the equilibrium properties of *δ*-Pu presented in this work would mark an important step towards an accurate description of more complicated problems in terms of large-scale atomistic simulations.
